# Electrical Impedance Tomography, Artificial Intelligence, and Variable Ventilation: Transforming Respiratory Monitoring and Treatment in Critical Care

**DOI:** 10.3390/jpm14070677

**Published:** 2024-06-24

**Authors:** Iacopo Cappellini, Lorenzo Campagnola, Guglielmo Consales

**Affiliations:** Department of Critical Care, Section of Anesthesiology and Critical Care, Azienda USL Toscana Centro, Ospedale Santo Stefano, 59100 Prato, Italy; lorenzo.campagnola@uslcentro.toscana.it (L.C.); guglielmo.consales@uslcentro.toscana.it (G.C.)

**Keywords:** EIT, ARDS, variable ventilation, artificial intelligence, critical care

## Abstract

Background: Electrical Impedance Tomography (EIT), combined with variable ventilation strategies and Artificial Intelligence (AI), is poised to revolutionize critical care by transitioning from reactive to predictive approaches. This integration aims to enhance patient outcomes through personalized interventions and real-time monitoring. Methods: this narrative review explores the principles and applications of EIT, variable ventilation, and AI in critical care. EIT impedance sensing creates dynamic images of internal physiology, aiding the management of conditions like Acute Respiratory Distress Syndrome (ARDS). Variable ventilation mimics natural breathing variability to improve lung function and minimize ventilator-induced lung injury. AI enhances EIT through advanced image reconstruction techniques, neural networks, and digital twin technology, offering more accurate diagnostics and tailored therapeutic interventions. Conclusions: the confluence of EIT, variable ventilation, and AI represents a significant advancement in critical care, enabling a predictive, personalized approach. EIT provides real-time insights into lung function, guiding precise ventilation adjustments and therapeutic interventions. AI integration enhances EIT diagnostic capabilities, facilitating the development of personalized treatment plans. This synergy fosters interdisciplinary collaborations and sets the stage for innovative research, ultimately improving patient outcomes and advancing the future of critical care.

## 1. Introduction

Electrical Impedance Tomography (EIT) has emerged as a significant innovation, particularly in critical care where it has a transformative impact [1]. EIT, grounded in the principle of impedance sensing, exploits the distinct electrical properties of tissues to create dynamic images of internal physiology without the use of ionizing radiation. Despite advancements, the integration of EIT with variable ventilation and Artificial Intelligence (AI) remains underexplored. This narrative review aims to analyze the convergence of these technologies, examining their current applications and future potential in critical care. The review highlights gaps in existing research, proposing a pathway for enhanced patient outcomes through personalized and real-time monitoring.

## 2. Methods

This narrative review is based on an extensive literature search conducted using databases such as PubMed, Google Scholar, and IEEE Xplore. Keywords included “Electrical Impedance Tomography”, “Artificial Intelligence”, “variable ventilation”, and “critical care”. Articles were selected based on relevance, with a focus on recent advancements and clinical applications. The selected literature was categorized into sections on EIT technology principles, AI integration, and variable ventilation techniques. This approach ensures a comprehensive analysis of the current state of research and its future directions.

## 3. The Basics of EIT

### 3.1. Impedance Sensing in EIT

At the core of EIT lies the measurement of electrical impedance, (Z), a complex number comprising resistance (R) and reactance (X), represented by the equation (Z=R+jX). This impedance reflects the opposition that tissues offer to the electrical current flow, varying distinctly among different tissue types due to their unique cellular structures and electrolyte contents. The lungs, with their air-filled alveoli interspersed with vascular structures, exhibit an impedance distinctly different from that of muscular or osseous tissues [2]. EIT capitalizes on this disparity by administering minute, non-hazardous electrical currents through electrodes placed around the thorax and measuring the resulting voltage changes to deduce the impedance map within the target area.

**Figure 1:**
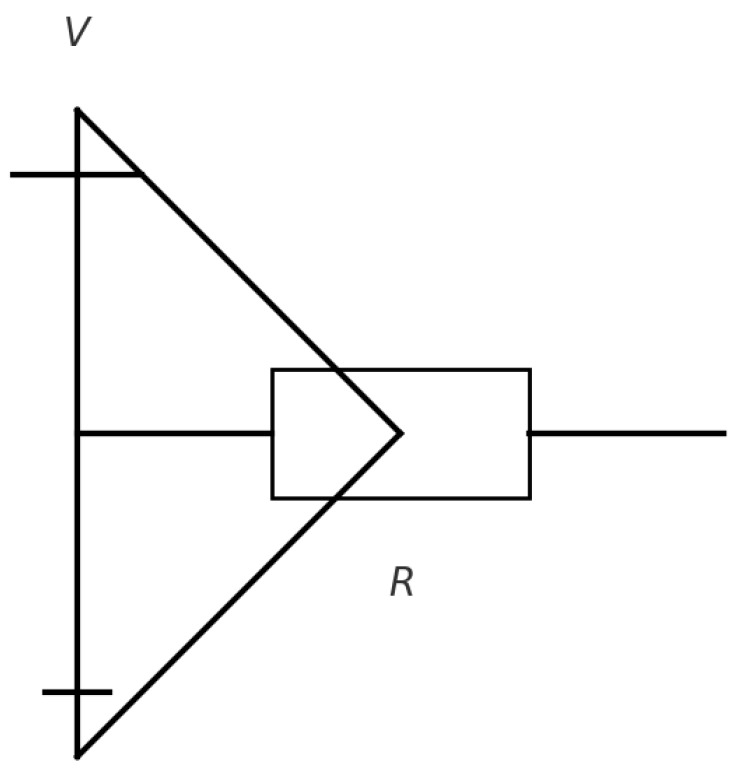
A conductivity circuit comprising a voltage source, represented as (V), and a resistor, denoted as (R). Here, the resistor represents the material’s resistance to an electrical current flow. Conductivity, symbolized as (σ), measures the ease with which an electric current can navigate through a material. Analogous to circuitry, this concept is depicted through a simple resistor, encapsulating the principle that higher conductivity equates to lower resistance. The mathematical relation linking conductivity and resistivity, represented as (ρ), is given by [σ=1/ρ]. This relationship indicates that materials characterized by high conductivity present a low resistance, thereby facilitating the passage of an electric current. Conversely, materials with low conductivity demonstrate a higher resistance, thus hindering electrical flow.

**Figure 2:**
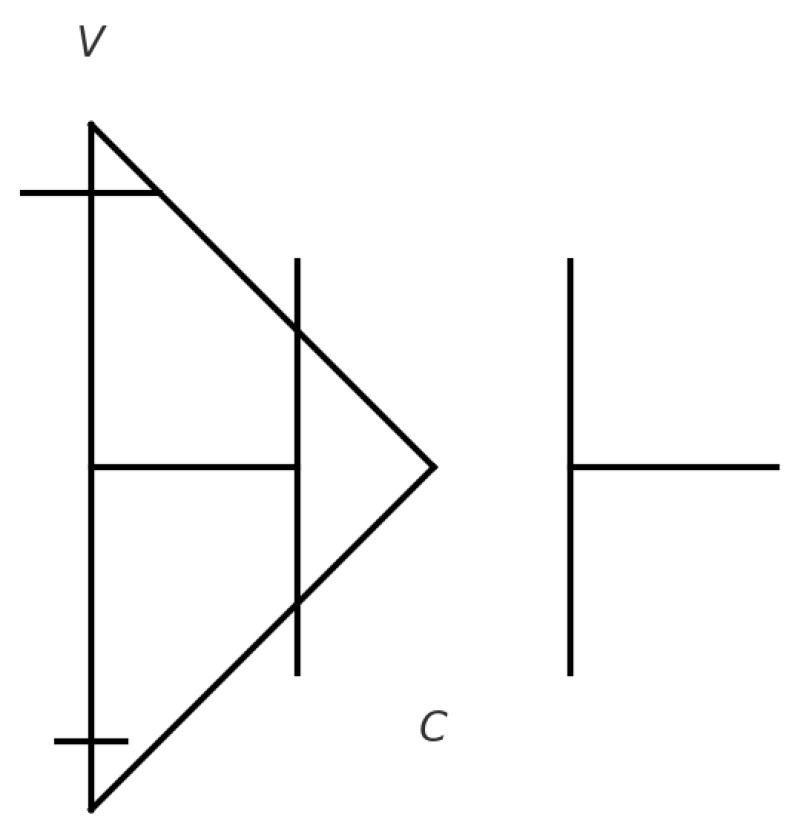
A permittivity circuit with a voltage source (V) and a capacitor (C). The capacitor represents a material’s ability to store electrical energy as an electric field, directly related to its permittivity, (ε). Permittivity measures a material’s capacity to support an electric field. In circuits, capacitors illustrate this trait, with materials of higher permittivity able to store more electric charge, leading to greater capacitance. The relationship between permittivity and a parallel-plate capacitor’s capacitance is given by [C=ε (A/d)], where (A) is the plate area and (d) is the plate separation. This formula shows how capacitance increases with the capacitor’s size and its permittivity.

The sensitivity of EIT to the conductivity (σ) and permittivity (ϵ) of tissues enhances its imaging capability. Conductivity, the tissue’s ability to carry electric current, is described by the relationship σ=J/E, where (J) is the current density and (E) is the electric field at a given location (Figure 1). Permittivity represents how an electric field interacts with a dielectric medium and varies with the applied electric field’s frequency (Figure 2). The unique conductive and permittivity properties of biological tissues at different frequencies enable EIT to distinguish between various tissue types, states, and changes such as the presence of air or fluid in the lungs [3].

### 3.2. EIT Image Reconstruction

**Figure 3:**
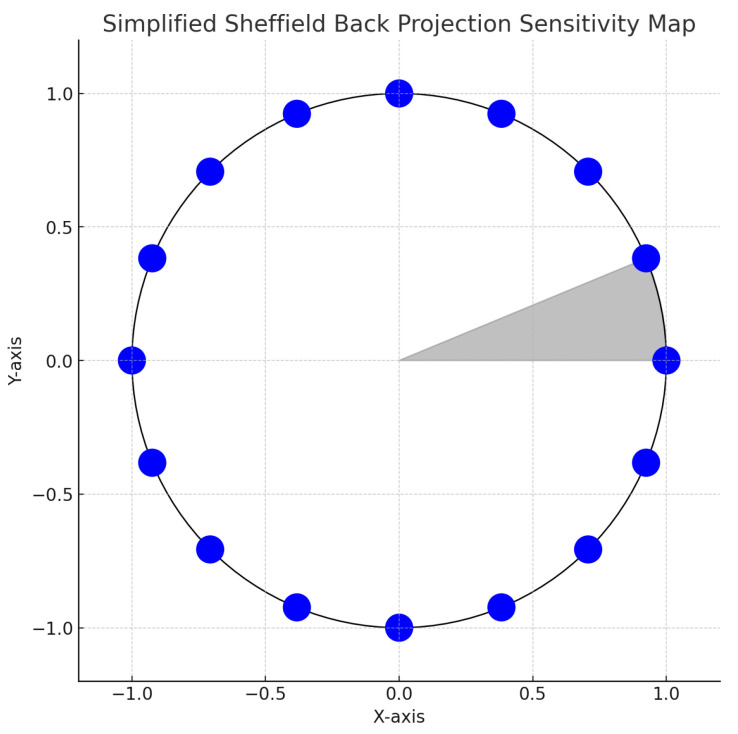
This figure illustrates the Sheffield back-projection algorithm for EIT, a pioneering method developed at the University of Sheffield for EIT image reconstruction. It simplifies the process by representing the object’s cross-sectional boundary as a circle, with the blue circles indicating the electrodes around this boundary. A grey wedge shows the sensitivity map, highlighting the regions most affected by voltage changes due to current injection. This algorithm, though less precise than contemporary iterative methods, offers a straightforward and quick solution for real-time imaging, making it invaluable for educational purposes and initial analysis. It operates by placing electrodes around the subject, applying alternating current, measuring voltage differences, and then using those measurements to generate an image through back-projection and filtering to counteract blurring. Despite its simplicity, modern techniques offering higher resolution and accuracy through advanced modeling have largely superseded it, yet its educational value and historical significance remain.

**Figure 4:**
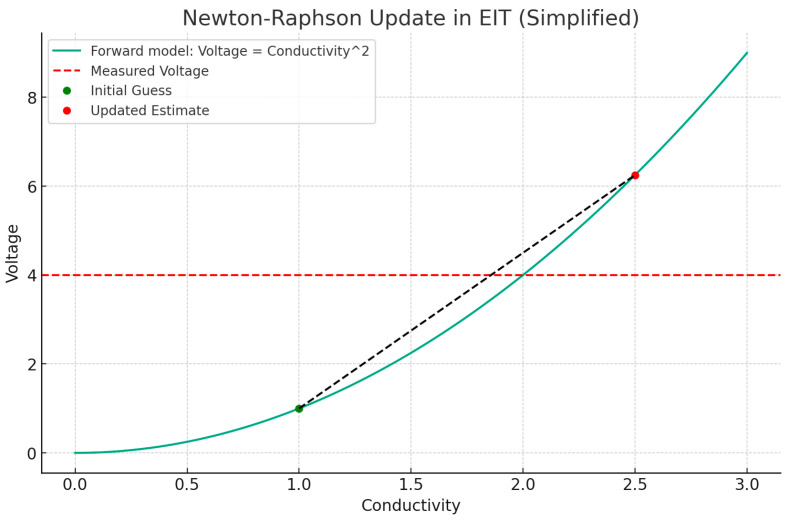
This figure demonstrates the Newton–Raphson method in EIT through a simplified one-dimensional example. It illustrates the method’s iterative process of updating an initial conductivity estimate toward a more accurate value. Key elements include a forward model curve predicting voltage from conductivity, a measured voltage line indicating true conductivity, and markers for the initial and updated estimates. The black dashed line traces the updated path from the initial guess to a closer approximation of true conductivity, highlighting the method’s effectiveness in reducing discrepancies between predicted and measured voltages for refining EIT conductivity images.

**Figure 5:**
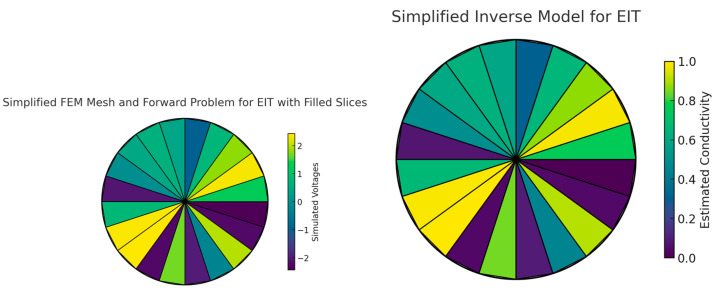
The images illustrate a simplified approach to EIT reconstruction using the Graz reconstruction algorithm. Initially, a finite element method (FEM) mesh, assuming uniform conductivity, models the area under study, such as a thorax cross-section. This model assigns voltages based on proximity to simulate a forward problem. Subsequently, an ‘inverse problem’ step recalibrates the mesh’s conductivity to reflect new measured voltages, incorporating simulated noise to mirror real conditions. This results in an updated conductivity map, visualizing the internal distribution inferred from external measurements. The Graz algorithm tackles the reconstruction through iterative solutions, addressing the inverse problem’s complexity and the ill-posed nature of estimating internal conductivity from surface measurements. It involves advanced mathematical techniques and regularization strategies to stabilize the solution against inaccuracies. The process begins with voltage predictions at electrode boundaries, based on an assumed internal conductivity distribution, and iteratively refines this estimate to minimize discrepancies between predicted and measured voltages. This iterative reconstruction culminates in a visual representation of spatial conductivity variations, offering insights into physiological aspects like lung ventilation or perfusion. The Graz algorithm incorporation of electrode behavior models and mathematical optimizations enhances reconstruction accuracy, proving essential for real-time, clinical monitoring of physiological parameters. Developing and applying such sophisticated algorithms requires a deep knowledge of numerical methods, inverse problem-solving, and physiology, often facilitated by specialized software integrated with EIT hardware.

The process of reconstructing images in EIT is a sophisticated endeavor that transforms measured voltage fluctuations into coherent images depicting the body’s conductivity distribution. This task is accomplished via intricate algorithms designed to address the inverse problem, i.e., deducing the internal conductivity from surface voltage readings. These algorithms navigate the complexities posed by the limited data available, attributable to the finite count of electrodes and the intricate three-dimensional structure of the human anatomy. A range of reconstruction methodologies exist, spanning from linear methods known for their computational efficiency—albeit with potential compromises in accuracy—to iterative algorithms that enhance the precision of reconstructions through progressive refinements. Notable techniques, including the Sheffield back-projection algorithm, Newton–Raphson iterative methods, the Graz reconstruction algorithm, along with contemporary enhancements of optimization through particle swarm and deep learning approaches, encapsulate the broad array of strategies utilized in EIT image reconstruction (Figure 3, Figure 4 and Figure 5) [4,5,6].

These algorithms work in tandem with the hardware components of EIT systems, including current injectors, voltage sensors, multiplexers, and data acquisition systems, to produce images that can be interpreted by clinicians. This process enables the visualization of dynamic physiological events such as pulmonary ventilation and perfusion, providing valuable real-time feedback for respiratory care management [4,7].

The Newton–Raphson method in EIT is used to update the conductivity estimate iteratively by minimizing the difference between the measured boundary voltages and the boundary voltages predicted by a forward model based on the current conductivity estimate [8]. A graphical representation of this process involves:

1. Forward model: computation of the predicted boundary voltages from an initial guess of the conductivity distribution.
2. Jacobian matrix: calculation of the sensitivity of the boundary voltages to changes in conductivity, known as the Jacobian or sensitivity matrix.
3. Mismatch: determination of the mismatch between the predicted and measured voltages.
4. Update: use of the Newton–Raphson method to update the conductivity distribution based on the mismatch and the Jacobian matrix [9].

The integration of contrast agents into EIT markedly enhances its ability to visualize and quantify pulmonary perfusion, thereby augmenting the modality’s diagnostic capabilities. Introducing a contrast agent, such as hypertonic saline, with electrical impedance properties distinct from blood temporarily alters the electrical characteristics within the pulmonary vasculature. EIT sensitivity to these changes facilitates the mapping of lung blood flow patterns, offering a novel approach to the assessment of pulmonary perfusion.

This advancement is complemented by innovative dynamic filtering techniques, including principal component analysis and frequency domain filtering, which effectively segregate cardiac influences from pulmonary signals in EIT data. This real-time separation, achieved without the need for breath holding or ECG gating, is pivotal for the precise visualization of perfusion patterns, distinguishing blood flow signals from those related to ventilation. Employing a first-pass kinetics approach, where a bolus of hypertonic saline serves as the contrast medium during a brief respiratory pause, enhances EIT utility in lung perfusion assessment. The passage of this bolus through the pulmonary vasculature, given its higher conductivity relative to blood, generates a discernible signal within the EIT data. This facilitates a direct evaluation of perfusion anomalies and regional blood flow analysis, thereby improving the accuracy of perfusion imaging. These methodologies, whether utilizing ECG gating or principal-component-analysis-based algorithms, refine EIT data to mitigate the impact of heart-related impedance changes on the interpretation of perfusion metrics. 

The adoption of EIT with contrast agents signifies a leap toward non-invasive, bedside monitoring of lung perfusion, offering insights into the ventilation–perfusion ratio. This ratio is crucial for diagnosing conditions like pulmonary embolism or edema and for assessing the efficacy of treatments, such as mechanical ventilation. Advancements in EIT technology and methods, as developed by Frerichs and colleagues, promise more detailed and ongoing analysis of lung function in clinical environments, underscoring the potential of EIT in revolutionizing pulmonary diagnostics and care [10,11].

## 4. EIT and ARDS: Unveiling Pulmonary Inhomogeneities

EIT represents a transformative advancement in the management of Acute Respiratory Distress Syndrome (ARDS), a condition characterized by acute hypoxemic respiratory failure and non-cardiogenic pulmonary edema [12]. The complexity of ARDS, with its diverse lung injury patterns, necessitates precise, real-time imaging to guide therapeutic decisions and interventions [13,14].

Here is a more detailed examination of how EIT is enhancing ARDS management across several crucial dimensions:

- Detection of regional lung function: EIT has proved to be indispensable for identifying specific lung areas affected by overdistension and recruitment. By providing a clear image of these regions, EIT enables clinicians to tailor ventilatory support accurately, addressing the heterogeneity of lung damage that typifies ARDS. This capability is critical, as it helps avoid the application of uniform ventilatory pressures across varied lung conditions, thus optimizing individual patient care [10].
- The effectiveness of ARDS treatments, such as Positive End-Expiratory Pressure (PEEP) and prone positioning, significantly depends on the timely and accurate assessment of their impact on lung mechanics. EIT offers a unique advantage in this regard, providing a direct, real-time view of how these interventions affect lung ventilation and perfusion. This capability allows clinicians to make informed decisions and adjustments based on immediate feedback from the patient’s respiratory status. For example, research by Songsangvorn et al. has demonstrated that EIT-guided PEEP titration can significantly enhance lung compliance [14]. By using EIT to tailor PEEP settings to the individual patient’s needs, clinicians can ensure that the applied PEEP levels are neither too high, which could lead to overdistension, nor too low, which might result in insufficient alveolar recruitment. This personalized approach helps optimize the balance between lung recruitment and the avoidance of overdistension, thereby improving overall respiratory mechanics. Moreover, EIT-guided PEEP titration has been shown to reduce mechanical power and the driving pressures required for ventilation. Mechanical power is a critical parameter, as excessive mechanical energy delivered to the lungs can exacerbate Ventilator-Induced Lung Injury (VILI). By lowering the mechanical power, EIT-guided interventions can mitigate the risk of VILI, contributing to better lung protection and potentially improving patient outcomes. Several studies corroborate these findings. A randomized controlled trial conducted at the First Medical Centre of Chinese PLA General Hospital involved patients undergoing robotic-assisted laparoscopic surgery. The study found that individualized EIT-titrated PEEP significantly improved respiratory system compliance, reduced driving pressures, and enhanced oxygenation compared to traditional fixed PEEP settings. Additionally, the incidence of postoperative atelectasis was markedly lower in the EIT group, underscoring the clinical benefits of EIT-guided PEEP adjustments [15]. In another study by Jimenez et al., EIT-guided PEEP titration was compared to PEEP selection using the High-PEEP/FiO_2_ table in patients with moderate-to-severe ARDS. The findings indicated that EIT-guided PEEP significantly reduced mechanical power and driving pressure, improved static respiratory system compliance, and decreased peak and plateau pressures. These results suggest that EIT-guided PEEP titration is more effective in optimizing lung-protective ventilation and reducing VILI [16]. Overall, the integration of EIT in monitoring and guiding therapeutic interventions such as PEEP and prone positioning represents a significant advancement in the management of ARDS. By providing real-time, detailed insights into lung mechanics, EIT enables a more precise and individualized approach to ventilation, thereby enhancing the effectiveness of treatments and improving patient outcomes [14,15,17,18,19].
- Optimizing mechanical ventilation: EIT’s ability to provide immediate feedback on lung mechanics is crucial for the fine-tuning of mechanical ventilation settings. This feedback helps clinicians balance the need for adequate oxygenation with the risk of exacerbating lung injury, thus significantly impacting patient prognosis. By adjusting ventilation parameters in response to real-time data, EIT helps mitigate the risk of VILI, a common complication in ARDS treatment [20].
- Guiding recruitment maneuvers: recruitment maneuvers are useful for managing ARDS as they help open up collapsed alveoli, potentially improving oxygenation and reducing the overall risk of barotrauma. EIT’s instant feedback during these maneuvers guides clinicians in adjusting their techniques to achieve optimal results without overdistending the lungs, thereby refining the management strategy for ARDS [21].
- Integrating concepts of Patient Self-Inflicted Lung Injury (P-SILI) and the Macklin effect: EIT is instrumental in monitoring the risk factors associated with P-SILI, where patients’ spontaneous efforts can exacerbate lung injury, and with the Macklin effect, which involves air leakage along bronchovascular sheaths following alveolar rupture. This monitoring is critical for spontaneously breathing ARDS patients, where elevated intrathoracic pressures can lead to significant complications. EIT’s capability to detect early signs of these phenomena allows for prompt and effective interventions, preserving lung integrity and optimizing outcomes [22].

**Figure 6:**
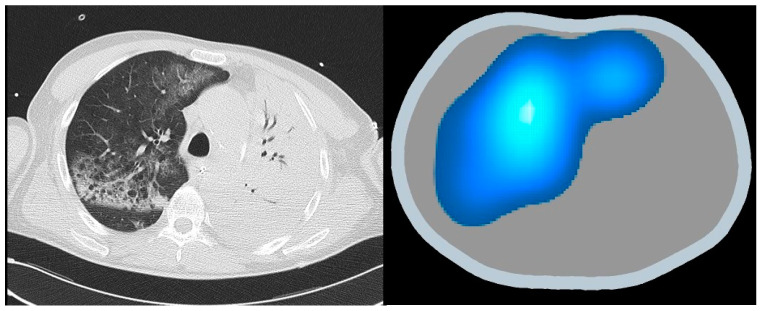
Revealing lung inhomogeneities in ARDS: comparing CT scan with EIT.

The incorporation of EIT within the schema of ARDS management heralds a paradigmatic shift toward a more individualized, data-driven ethos of care. This integration not only fosters a more profound comprehension of the pathophysiology underpinning ARDS, but also augments the avenue for personalized therapeutic stratagems. The confluence of EIT’s real-time visualization and the data-driven insights it engenders marks a new era in the quest for a more refined and effective management of ARDS. Through the lens of EIT, the veil shrouding pulmonary inhomogeneities in ARDS is gradually lifted, thus offering a clearer sight for tailored therapeutic interventions (Figure 6).

## 5. Synergy with Variable Ventilation

### 5.1. Physiology of Respiratory Variability

Variable ventilation mimics the natural variability of human breathing, which is evident in the healthy lung’s response to different physiological states. This variability is characterized by changes in tidal volume and respiratory rate, enhancing the respiratory system’s efficiency and adaptability. In pathophysiological states, such as ARDS or COPD, this inherent variability is significantly reduced, often leading to compromised lung function. The physiological rationale for introducing variability in mechanical ventilation is based on the premise that it can restore some of the natural dynamics of the respiratory system, potentially leading to improved lung recruitment and gas exchange [17,23,24].

### 5.2. Technique of Mechanical Ventilation

Variable Mechanical Ventilation (VMV) employs alternating levels of tidal volumes and respiratory rates, unlike conventional mechanical ventilation which delivers set, uniform breaths. VMV aims to replicate the natural variability seen in spontaneous breathing, which may involve using patterns that distribute tidal volumes based on a Gaussian or power-law distribution. This approach is hypothesized to improve alveolar recruitment, reduce cyclic atelectasis, and minimize Ventilator-Induced Lung Injury (VILI) by preventing the overdistension and repetitive opening and closing of alveolar units [23,24].

### 5.3. Potential Role of EIT in Guiding Variable Ventilation

EIT, a non-invasive monitoring technique, can significantly enhance the application of VMV by providing real-time images of regional lung ventilation. By visualizing how air distributes across the lung, EIT can help clinicians optimize ventilator settings to ensure a more uniform lung aeration and reduce the risk of VILI. EIT’s ability to assess regional lung function dynamically makes it an invaluable tool to adjust variable ventilation protocols to individual patient needs, thus enhancing the personalized approach in mechanical ventilation. In clinical practice, integrating EIT with variable ventilation could potentially transform patient outcomes in critical care by enabling a more precise management of ventilatory support, particularly in patients with heterogeneous lung diseases. The synergy between EIT and variable ventilation could lead to improved strategies for the management of ARDS, where lung protection and optimization of gas exchange are crucial [23].

The integration of EIT into the management of variable ventilation represents a significant advancement in the personalized care of patients requiring mechanical ventilation. By leveraging the detailed, real-time data provided by EIT, clinicians can not only tailor ventilation more closely to the physiological needs of the patient, but also potentially improve outcomes by minimizing lung injury and enhancing overall lung function [25].

## 6. Harnessing the Power of Artificial Intelligence

The integration of Artificial Intelligence (AI) with Electrical Impedance Tomography (EIT) is continuously advancing, leading to an increasing number of applications in medical imaging and diagnostics. This chapter delves into the profound impact AI has on enhancing EIT, exploring the sophisticated techniques and innovative approaches that are shaping the future of this technology.

### 6.1. Advanced Neural Network Applications in EIT

Deep learning techniques have been pivotal to significantly improve the quality and resolution of EIT image reconstruction. Researchers have explored various methods to enhance EIT imaging. One such method is single network reconstruction, where individual neural networks are utilized to directly reconstruct images from EIT data. These networks are capable of learning complex relationships between electrical measurements and internal conductivity distributions, thereby producing high-resolution images that are crucial for accurate diagnostics [26,27,28].

For instance, the study by Yang et al. investigated the application of various machine learning models to predict the outcomes of High-Flow Nasal Cannula (HFNC) therapy using EIT-derived image features. The researchers utilized methods such as discriminant analysis, ensemble methods, K-Nearest Neighbors (KNN), Artificial Neural Networks (ANN), Support Vector Machines (SVM), AdaBoost, XGBoost, logistic regression, random forest, Bernoulli Bayes, Gaussian Bayes, and Gradient-Boosted Decision Trees (GBDT) to build prediction models. Among these, XGBoost demonstrated a superior performance, highlighting its suitability for early prediction of HFNC outcomes based on EIT data [29]. The integration of AI with EIT involves several key steps:

Feature extraction: EIT data are processed to extract significant features such as Global Inhomogeneity (GI), Center of Ventilation (CoV), Regional Ventilation Delay (RVD), Tidal Impedance Variation (TIV), and End-Expiratory Lung Impedance (EELI). These features are critical to assess lung function and predict patient outcomes [30].

Data preprocessing: techniques like Z-score standardization and Synthetic Minority Oversampling Technique (SMOTE) are employed to normalize the data and address class imbalances, thereby improving the robustness and accuracy of the prediction models [31].

Model training and validation: machine learning models are trained on the preprocessed EIT data and their performance is validated using metrics such as accuracy, recall, precision, specificity, and Area Under the Curve (AUC). The study by Yang et al. found that data balancing significantly improved the specificity of the models, with XGBoost showing the best overall performance [29].

The synergy between variable ventilation strategies and real-time EIT monitoring can optimize lung recruitment and minimize Ventilator-Induced Lung Injury (VILI). By leveraging AI, clinicians can dynamically adjust ventilation settings based on real-time EIT data, ensuring optimal patient-specific treatment.

Studies like the previous one underscore the potential of integrating machine learning with EIT to develop predictive models that can aid clinical decision-making. These models can provide early warnings of treatment failure, allowing for timely interventions and potentially improving patient outcomes in critical care settings.

Another approach involves the integration of these neural networks with traditional algorithmic EIT reconstruction techniques. By combining deep learning models with established EIT algorithms, researchers have developed synergistic methods that leverage the strengths of both approaches. This integration results in more robust and accurate image reconstructions, enhancing the clinical utility of EIT.

Furthermore, hybrid systems that combine multiple neural networks have been developed to tackle the challenges of EIT imaging. These systems enhance image quality by optimizing various aspects of the reconstruction process, such as measurement techniques and electrode placements. This holistic approach ensures that EIT can be effectively used for real-time monitoring and diagnostics, offering a significant improvement over traditional methods [26,27,28].

### 6.2. Challenges and Innovations

Despite these advances, the field faces several challenges. One of the primary issues is the need for large, high-quality datasets to effectively train AI models. Collecting and annotating such datasets is both resource-intensive and time-consuming. Additionally, integrating AI with existing medical technologies poses technical challenges related to data compatibility, processing speeds, and hardware limitations.

To address these challenges, researchers have turned to innovative solutions such as generative models. Generative Adversarial Networks (GANs), for example, have been utilized to create synthetic data. This approach allows for the generation of extensive datasets that can be used to train AI models, thereby enhancing the predictive power of EIT without the constraints posed by small or inconsistent datasets [28,32,33]. By generating realistic bioimpedance data, GANs facilitate the development of more accurate and reliable EIT models, ensuring that the technology can be effectively used in clinical settings.

Moreover, reinforcement learning algorithms have shown promise in clinical decision support systems. These algorithms can dynamically adjust EIT settings based on real-time patient data, optimizing the balance between image quality and patient safety. This adaptive capability is crucial for personalized medicine, where treatment protocols need to be tailored to the individual needs of each patient [34].

### 6.3. Comparative Analysis and Mesh Optimization

Significant efforts have been made to optimize neural network training using refined and coarse mesh configurations. This optimization aims to reduce discretization errors, which are common in EIT image reconstruction. By training neural networks with inputs from a refined mesh and outputs defined by a coarser mesh, researchers have achieved a balance between computational efficiency and image fidelity.

Bianchessi et al. have conducted extensive comparative analyses of various neural network architectures, such as the LeNet model and fully connected feed-forward networks, to determine their efficacy in different EIT applications. Their findings indicate that certain configurations perform better under specific conditions, which is critical for tailoring AI strategies to meet the unique demands of EIT. These analyses are instrumental in identifying the most effective AI strategies for enhancing EIT applications, providing a roadmap for future research and development [35].

### 6.4. Digital Twins in Medical Imaging

The concept of digital twins represents a groundbreaking advancement in the integration of AI with EIT. Digital Twins (DTs) create virtual replicas of patients’ lungs, incorporating both biomechanical and electrical properties to simulate and predict physiological responses under various scenarios. As explored by Jiang et al., this technology, when combined with EIT, offers a dynamic and precise method for lung monitoring [36]. Digital twins can provide real-time, patient-specific data that enhance the accuracy of diagnostic and therapeutic interventions, paving the way for truly personalized medicine. DTs further augment the capabilities of EIT by creating accurate virtual models of patients’ lungs. These models can simulate various physiological conditions, providing dynamic, real-time data that allows for personalized treatment plans [37]. The integration of DTs with EIT facilitates a deeper understanding of lung pathophysiology, enabling clinicians to anticipate and react to changes in patients’ conditions more effectively.

In conclusion, the integration of AI with EIT, spearheaded by studies from Hou et al., Zhang et al., Chen et al., and Bianchessi et al., represents a significant advancement in medical imaging. This robust framework not only improves the resolution and accuracy of EIT imaging, but also extends its applications to predictive diagnostics and personalized medicine. The ongoing innovations in AI are set to offer more effective, tailored patient care, heralding a new era of medical technology integration [38,39,40].

The collective efforts of these researchers are setting the stage for EIT to become a more powerful tool in medical imaging. By providing clinicians with faster, more accurate, and adaptively responsive imaging solutions, AI-enhanced EIT promises to revolutionize how respiratory conditions and other medical issues are diagnosed and monitored. As these technologies continue to evolve, they will undoubtedly lead to significant improvements in patient outcomes and the overall quality of healthcare [26,27,34,35,41,42,43,44,45,46,47,48]. Moreover, interdisciplinary collaboration will be crucial in advancing the integration of AI, EIT, and DTs technologies, fostering innovation that can transform critical care.

## 7. Conclusions—Revolutionizing the Art of Critical Care

EIT represents a pivotal innovation in critical care, offering a non-invasive, radiation-free modality for dynamic imaging of internal physiology. This review elucidates the foundational principles of EIT, emphasizing its reliance on impedance sensing to distinguish tissue types based on their electrical properties. The integration of EIT with variable ventilation and AI underscores a transformative shift toward predictive and personalized healthcare.

EIT utility in managing ARDS is profound, providing real-time insights into regional lung function, guiding therapeutic interventions, and optimizing mechanical ventilation. The technology’s capability to reveal pulmonary inhomogeneities in ARDS enhances individualized care strategies, facilitating precise adjustments to ventilation settings and improving patient outcomes.

Variable ventilation, mirroring natural respiratory variability, synergizes with EIT to refine ventilatory support, minimize VILI, and enhance gas exchange. EIT’s real-time imaging facilitates the dynamic adjustment of ventilation protocols, ensuring tailored treatment for heterogeneous lung conditions.

AI integration significantly advances EIT by enhancing image reconstruction quality through deep learning techniques, optimizing neural network configurations and leveraging generative models for data augmentation. The development of DTs further augments EIT, offering virtual patient replicas that simulate physiological responses, thereby refining diagnostic and therapeutic approaches.

In conclusion, this review uniquely combines the latest advancements in EIT with the transformative potential of Artificial Intelligence (AI) and variable ventilation strategies in critical care. Unlike previous reviews, we provide a comprehensive analysis of the synergistic integration of these technologies, demonstrating how EIT can be enhanced through AI to offer real-time, high-resolution imaging for precise lung monitoring. This integration not only improves diagnostic accuracy, but also enables personalized therapeutic interventions, optimizing patient outcomes in critical care settings.

## Data Availability

Not applicable.
